# Hybrid Approach of Aortic Diseases: Zone 1 Delivery and Volumetric Analysis on the Descending Aorta

**DOI:** 10.21470/1678-9741-2017-0040

**Published:** 2017

**Authors:** José Augusto Duncan, Ricardo Ribeiro Dias, Fabrício José Dinato, Fábio Fernandes, Félix José Álvares Ramirez, Charles Mady, Fabio Biscegli Jatene

**Affiliations:** 1 Instituto do Coração do Hospital das Clínicas da Faculdade de Medicina da Universidade de São Paulo (InCor-HCFMUSP), São Paulo, SP, Brazil.

**Keywords:** Aorta, Thoracic, Aneurysm, Dissecting, Minimally Invasive Surgical Procedures

## Abstract

**Introduction:**

Conventional techniques of surgical correction of arch and descending aortic diseases remains as high-risk procedures. Endovascular treatments of abdominal and descending thoracic aorta have lower surgical risk. Evolution of both techniques - open debranching of the arch and endovascular approach of the descending aorta - may extend a less invasive endovascular treatment for a more extensive disease with necessity of proximal landing zone in the arch.

**Objective:**

To evaluate descending thoracic aortic remodeling by means of volumetric analysis after hybrid approach of aortic arch debranching and stenting the descending aorta.

**Methods:**

Retrospective review of seven consecutive patients treated between September 2014 and August 2016 for diseases of proximal descending aorta (aneurysms and dissections) by hybrid approach to deliver the endograft at zone 1. Computed tomography angiography were analyzed using a specific software to calculate descending thoracic aorta volumes pre- and postoperatively.

**Results:**

Follow-up was done in 100% of patients with a median time of 321 days (range, 41-625 days). No deaths or permanent neurological complications were observed. There were no endoleaks or stent migrations. Freedom from reintervention was 100% at 300 days and 66% at 600 days. Median volume reduction was of 45.5 cm^3^, representing a median volume shrinkage by 9.3%.

**Conclusion:**

Hybrid approach of arch and descending thoracic aorta diseases is feasible and leads to a favorable aortic remodeling with significant volume reduction.

**Table:** 

Abbreviations, acronyms & symbols	
TEVAR	= Thoracic endovascular aortic repair

## INTRODUCTION

Conventional techniques for surgical correction of aortic arch and descending diseases (either aneurysms or dissections) requires extracorporeal circulation associated with deep hypothermia and circulatory arrest. It remains as high-risk procedures, although recent advances (pre-, intra- and postoperatively) have improved results dramatically^[1]^.

Endovascular treatments of abdominal and thoracic descending aorta are already well-established and have a lower surgical risk when compared to conventional techniques^[2]^. Improvement of both, techniques and endoprosthesis, made endovascular management of more complex aortic diseases, like those affecting distal arch and proximal descending aortic segments also possible, reducing treatment morbimortality of the procedure^[3]^.

In order to make it feasible, sophisticated vascular techniques were improved aiming to keep cerebral perfusion. Debranching of supra-aortic vessels enables access to healthier aortic sections, propitious to serve as landing zones to endovascular prosthesis^[3,4]^. This hybrid aortic arch approach has emerged as a less invasive option, mainly to high-risk patients^[5,6]^.

Giving this background, the treatment addressed to these patients consists in performing, previously to the endoprosthesis implant, debranching of the supra-aortic vessels to obtain at least 2 cm of healthy aorta.

The purpose of this study is to evaluate the aortic remodeling after hybrid approach of the distal arch and proximal descending aortic diseases by means of volumetric analysis of the descending thoracic aorta.

## METHODS

### Patient Population

We performed a retrospective review of our single-center results of all patients who underwent hybrid aortic repair for complex aneurysms or type B dissection between September 2014 and August 2016. Inclusion criteria was related to any involvement of the distal aortic arch and proximal descending aortic disease with an inappropriate direct landing zone for Thoracic EndoVascular Aortic Repair (TEVAR). The landing zone chosen had to be zone 1. Patients with ascending or proximal arch pathology were excluded from the study. Data were prospectively collected for patient demographics, indications of intervention, risk factors, procedures and outcomes.

Seven consecutive patients underwent hybrid aortic repair, meaning double debranching of the supra-aortic vessels in order to create a suitable proximal landing zone to the endograft right after the origin of the innominate artery - according to Ishimaru and Mitchell classification, zone 1^[7]^.

All patients included in this study had computed tomography angiography pre- and postoperatively, allowing volume measurements and comparison.

### Indications for Intervention

Patients were referred to surgery according to the European guideline for aortic treatment^[8]^ - when the largest diameter reached 55 mm, growth rate over 0.5 cm/year or when the patient presented with symptoms.

In our series, the largest diameter ranged from 42.4 mm to 91 mm (median 60.5 mm). Only one patient had the largest diameter < 55 mm, a symptomatic type B chronic dissection. In total, two (28.6%) patients were symptomatic and five were asymptomatic (Table 1).

**Table 1:** Indications for hybrid aortic arch intervention.

Indications for intervention	n	%
Symptomatic	2	28.6
Chronic dissection	1	
Aneurysm	1	
Asymptomatic	5	71.4
Chronic dissection	2	
Aneurysm	3	

Considering the disease, patients with chronic dissection had diameters ranging from 42.4 mm to 61.3 mm while patients with aneurysm had diameters from 60.1 mm to 91 mm (Table 2).

**Table 2:** Diameters according to disease.

Disease	Diameters (mm), range
Chronic dissection	42.4-61.3
Aneurysm	60.1-91

### Debranching Procedure

All bypass procedures were performed in a hybrid operating room, with the patient under general anesthesia and receiving intravenous heparin.

Access to and isolation of supra-aortic vessels was achieved through upper ministernotomy (L-shaped, left second intercostal space). All patients were submitted to double debranching, in order to make possible to anchor the endoprosthesis at zone 1.

**Fig. 1:**
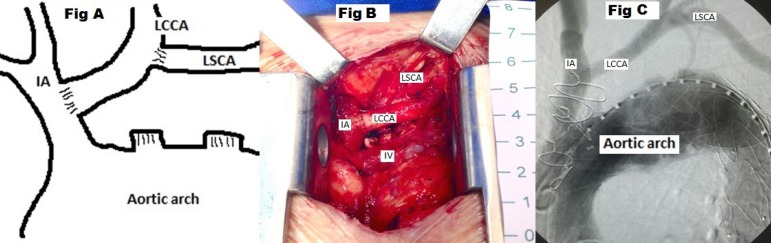
Debranching to zone 1: scheme (A), intraoperative picture (B) and angiography confirming patency and endograft position without endoleaks (C). IA=innominate artery; IV=innominate vein; LCCA=left common carotid artery; LSCA=left subclavian artery

Left subclavian artery was approached firstly, dissecting it as far distal as possible. It was then clamped, sectioned right after its origin and the proximal stump carefully closed with 4-0 or 5-0 monofilament sutures. An arteriotomy was made at the lateral side of the left common carotid artery and the end-to-side anastomosis was performed with a 5-0 monofilament suture without tension. Similar technique was used to anastomose the left common carotid artery to the innominate artery (Figures 1A, 1B and 1C).

Revascularization of the left subclavian artery was performed in every patient. When a graft was needed (to avoid a tensioned anastomosis), a 6-mm Dacron^®^ graft (Jotec Inc., Hechingen, Germany) was used either to achieve transposition of the left subclavian artery to the left carotid artery (one patient, 14.3%) or to bypass the left carotid artery to the innominate artery (two patients, 28.6%). An aortography was performed right after the debranching procedure to confirm the patency of all supraaortic vessels.

### TEVAR Procedure

All TEVAR procedures were performed as standardized TEVAR protocols. Access to the true lumen was obtained from a transfemoral approach after an inguinal cutdown. Through a 5F pigtail catheter, a 300-cm-long stiff wire (Lunderquist, Cook, Denmark) was placed at the ascending aorta using fluoroscopy. Another 5F pigtail catheter was placed through the contralateral femoral artery to the ascending aorta for repeated angiographies using 623 mg/mL iodine contrast (Ultravist 300; Bayer Pharma AG, Germany) during the procedure.

All stent grafts were deployed in the desired position (zone 1 delivery) under pharmacologically induced arterial hypotension, in order to avoid any windsock effect or misplacement.

Stent graft dimensions were chosen by measuring the diameter of the proximal and distal landing zone in an orthogonal view. This was possible after using a workstation to reformat the computed tomography angiography. In patients presenting with aortic dissection, no oversizing was done. Oversizing more than 20% was avoided when dealing with aneurysms. Balloon dilatation was only performed when a residual endoleak was noticed in dissections and always when treating aneurysms.

The three patients presenting with chronic type B aortic dissections were treated with one single endograft of 200 mm and the four other patients presenting with descending thoracic aorta aneurysms received up to three endografts, with a maximum coverage length of 330 mm (Table 3).

**Table 3:** Procedural data from TEVAR.

Intraoperative characteristics	n	%
Double debranching (zone 1)	7	100
Use of Dacron^®^ graft to debranch	3	42.9
LSCA-LCCA	1	
LCCA-IA	2	
One-stage procedure	6	85.7
Number of endoprosthesis		
1	5	71.4
2	1	14.3
3	1	14.3
Occlusion of left subclavian artery	__	__
Insertion through femoral artery	7	100
Proximal diameter of first endograft (median, mm)	38	(range 32-46)
Number of stents (median)	1	(range 1-3)
Total coverage length (median, mm)	200	(range 200-330)

IA=innominate artery; LCCA=left common carotid artery; LSCA=left subclavian artery

### Volume Measurement

All seven patients underwent a thin-cut (1- to 3-mm slices), electrocardiograph-gated computed tomography angiography both pre- and postoperatively. Axial static images were manipulated to multiplanar and tridimensional reconstructions in order to make more accurate measures. Those mensurations were made by the surgeons.

**Fig. 2:**
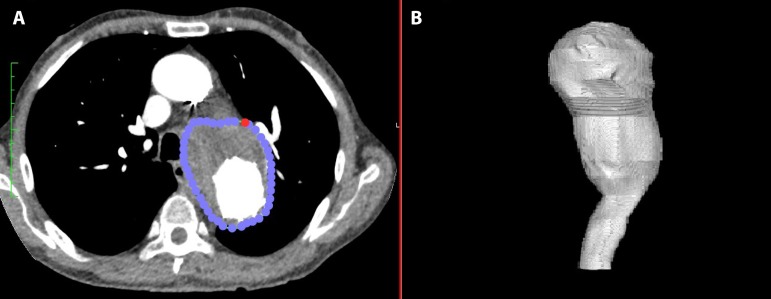
Steps of volume calculation: individually placed points delimitates the outer margin of total aorta at each axial image (A); resulting three-dimensional reconstruction (B).

On consecutive cross-sectional images, a series of individually placed points created an outline margin of the total aorta (Figure 2A). These delimitations were placed at every 1- to 3-mm distance from one another. Anatomical references for beginning and ending of the thoracic aorta were the left subclavian artery origin and celiac trunk origin. Volumetric measurements for the total descending thoracic aorta were then calculated as the sum of this series of irregular 1- to 3-mm-height cylinders (Figure 2B). All volumes were expressed in cm^3^.

### Follow-up

The protocol included a computed tomography angiography that was requested at first appointment after hospital discharge and annually thereafter.

### Definitions and Statistical Analysis

Technical success was defined as patency of supra-aortic vessels associated with correct position of the endograft and absence of endoleaks. Outcome criteria were defined according to the reporting standards for TEVAR^[9]^. The results were expressed as mean, median and standard deviations of continuous variables and frequencies and percentage frequencies of categoric factors. Analysis was done with Google Sheets (Google Inc., Palo Alto, CA, USA) and with Kaplan-Meier Survival Curve Grapher (Eureka Statistics - Peter Rosenmai, http://eurekastatistics.com/kaplan-meier-survival-curve-grapher/).

Measures and volume calculation pre- and postoperative were done through Horos software (Horos Project - DICOM image viewing and measuring. http://www.horosproject.org/). When patients had more than one postoperative computed tomography angiography, the last one was used.

## RESULTS

Preoperative patient characteristics and indications for hybrid aortic repair are described in Tables 1 and 4, respectively.

**Table 4:** Baseline characteristics of patients.

Characteristics	n	%
Mean age, ± SD, years	62.2±5.0	Range 55-68
Males	5	71.4
Hypertension	6	85.7
Diabetes	1	14.3
Hyperlipidemia	4	57.2
Chronic renal disease	__	__
Chronic renal disease, dialytic	__	__
Acute renal disease	__	__
Smoker	7	100
COPD	1	14.3
Family history	__	__
Dyspepsia	__	__
Stroke with deficits	__	__
Stroke without deficits	__	__
HIV	__	__
Cancer	1	14.3
Coronary artery disease	3	42.8
History of heart attack	1	14.3
Thoracic pain	2	28.5
Marfan syndrome	__	__
Bicuspid aortic valve	__	__
Atrial fibrillation	1	14.3
Alcohol abuse	2	28.5
Obesity (BMI > 30)	3	42.9
Elective	3	42.9
Urgent repair	4	57.2
Aneurysm	4	57.2
Acute type B dissection	__	__
Chronic type B dissection	3	42.9
Normal ventricular function (EF ≥ 55%)	6	85.7

BMI=body mass index; COPD=chronic obstructive pulmonary disease; EF=ejection fraction; HIV=human immunodeficiency virus; SD=standard deviation

Our population had hyperlipidemia in four (57.2%) patients, hypertension in six (85.7%) and history of smoking in all of them. Three (42.9%) patients were secondary of increasing in size of the aortic dissection and four (57.1%) secondary to aortic aneurysm. Two (28.5%) patients were symptomatic, referring thoracic pain - one had aneurysm and the other had chronic dissection.

Procedural data from TEVAR are summarized in Table 3. Two commercially available endoprosthesis were used, according to the availability in our institution. They comprised both Valiant Captivia (Medtronic, Minneapolis, MN, USA) in six (85.7%) patients and Gore TAG (W.L. Gore & Associates, Inc., Flagstaff, AZ, USA) in one (14.3%).

Follow-up was done in 100% of patients with a median time of 321 days (range 41-625 days). Technical success was achieved in all patients, with long-term patency of supra-aortic vessels, correct positioning of endoprosthesis and neither endoleaks nor migrations observed. Those items were documented through postoperative computed tomography angiography that was done at a median postoperative time of 286 days (range 38-572). There were no deaths.

Considering all complications following TEVAR in this study, one patient presented postoperatively with an aneurysm that grew in extension, despite of the absence of any visible endoleaks at the intraprocedural angiography. This growth was detected at the first postprocedural computed tomography angiography, performed after the outpatient return. We treated implanting a second endoprosthesis, increasing the coverage length. A computed tomography angiography performed 5 days postoperatively, before hospital discharge, demonstrated a reduction of 28.4 cm^3^, representing a decrease of 4.8%. This patient was the only one who required a reintervention, with freedom of reintervention in 100% at 300 days and 66% at 600 days.

Three (42.9%) patients presented with acute renal failure, defined in our study as a serum creatinine elevation of at least 0.5 mg/dL. None of them needed hemodialysis. No strokes with permanent deficits were detected, although one patient presented with a left facial paralysis that was completely resolved spontaneously after three days. A cranial computed tomography was done with no specific findings. There were also no permanent paraplegia even though one patient had transient symptoms attributed to medullary ischemia (lower limbs weakness and urinary retention). No major pulmonary problem was observed. No patients presented with retrograde type A dissection, access site complications, mesenteric ischemia or wound infections.

TEVAR was done simultaneously to the debranching procedure in 85.7% (6 patients). We performed a staged procedure in one patient because of some atherosclerotic plaques noticed at the debranching procedure. It was considered safer to observe if there would be any neurological deficit before the endograft implant.

Analysis of aortic morphology began with multiplanar and three-dimensional reconstruction to better define the anatomic limits to volume calculation of the total aorta. When comparing pre- and postoperative volumes it was noticed a median reduction of 45.5 cm^3^, representing a median shrinkage of 9.3%.

## DISCUSSION

It is known that the majority of thoracic aortic diseases are degenerative and occur in association with risk factors for atherosclerosis such as smoking, hypertension and hypercholesterolemia^[10]^ and all of those had a high prevalence in our sample.

One of the factors that impact morbimortality after aortic interventions are neurologic events and progression of aortic disease^[11]^. TEVAR is a safe and effective procedure to treat both aneurysms and dissections involving the descending aorta with relative low risk^[12,13]^. The landing zone was soon extended proximally in order to treat more extensive segments of the aorta, reducing the morbimortality when compared with conventional techniques^[14-16]^.

New totally endovascular techniques for treatment of complex aortic arch diseases are available and include stenting of the supra-aortic vessels (parallel techniques), fenestrated or branched endografts. Those are promising, but experience in aortic arch repair are very limited^[17]^ and we do not have them available for patients from the public health system.

Supra-aortic debranching is by itself a safe surgical procedure with a low complication rate^[18]^. In our study, no perioperative deaths, complications for local reasons or left recurrent laryngeal nerve injury occurred.

Stroke with permanent deficits did not occur in our patients. Nevertheless, it is a major concern since it can be the result of manipulation of the supra-aortic vessels while debranching or of embolism formation secondary to wire manipulation during TEVAR^[19]^. Spinal cord injury is also a significant problem, related to the extent of endograft coverage^[20]^. In our study, transient symptoms related to spinal cord ischemia happened to the patient with the largest coverage length (330 mm, from left carotid artery to just above the celiac trunk emergence).

Endoleaks after TEVAR are observed^[19]^, with up to 42% of incidence^[21]^. Although early endoleaks were not observed in our study, one patient had an aneurysm growth and needed a second procedure.

Reintervention was necessary in one (14.3%) patient after almost one year (321 days) and the technical success rate for this secondary procedure was 100%. This information highlights the need for a close surveillance in all patients after TEVAR, which is very challenging in a developing country with continental dimensions like Brazil.

At present, few data are available on volumetric outcomes of hybrid aortic procedures despite of its documented better sensitiveness to aneurysm size change when compared to diameter^[9,22]^. Volumetric calculation varies according to operator experience^[23]^ and although the median reduction in our series was found to be of 9.3%, there is a potential measurement error of 10%^[9]^. Time of follow-up must be also considered since remodeling continues up to five years^[22]^. When only patients with more than 250 days of follow-up were taken into account, median shrinkage rose to 13.5%. Furthermore, every patient of our study had thrombosis around the endoprosthesis, what has correlation with shrinkage^[22]^.

Despite the fact that this is a report of an initial experience in treating this extension of aortic disease and following up with volumetric analysis, it also must be said that the limitations of this study includes not only the reduced number of patients, but also the short follow-up time.

## CONCLUSION

Hybrid aortic zone 1 proximal delivery of endograft is a viable alternative to conventional aortic arch surgery in patients with both aneurysms and type B dissections. It leads to a favorable aortic remodeling that continues to improve over time. Further studies with a larger sample and longer follow-up are needed to confirm this idea.

**Table:** 

Authors' roles & responsibilities	
JAD	Agreement to be accountable for all aspects of the work in ensuring that questions related to the accuracy or integrity of any part of the work are appropriately investigated and resolved; final approval of the version to be published
RRD	Substantial contributions to the conception or design of the work; or the acquisition, analysis, or interpretation of data for the work; final approval of the version to be published
FJD	Substantial contributions to the conception or design of the work; or the acquisition, analysis, or interpretation of data for the work; final approval of the version to be published
FF	Drafting the work or revising it critically for important intellectual content; final approval of the version to be published
FJAR	Drafting the work or revising it critically for important intellectual content; final approval of the version to be published
CM	Drafting the work or revising it critically for important intellectual content; final approval of the version to be published
FBJ	Drafting the work or revising it critically for important intellectual content; final approval of the version to be published
